# The Spread of Bluetongue Virus Serotype 8 in Great Britain and Its Control by Vaccination

**DOI:** 10.1371/journal.pone.0009353

**Published:** 2010-02-22

**Authors:** Camille Szmaragd, Anthony J. Wilson, Simon Carpenter, James L. N. Wood, Philip S. Mellor, Simon Gubbins

**Affiliations:** 1 Pirbright Laboratory, Division of Epidemiology, Institute for Animal Health, Pirbright, Surrey, United Kingdom; 2 Cambridge Infectious Diseases Consortium, Department of Veterinary Medicine, University of Cambridge, Cambridge, Cambridgeshire, United Kingdom; University of Leeds, United Kingdom

## Abstract

**Background:**

Bluetongue (BT) is a viral disease of ruminants transmitted by *Culicoides* biting midges and has the ability to spread rapidly over large distances. In the summer of 2006, BTV serotype 8 (BTV-8) emerged for the first time in northern Europe, resulting in over 2000 infected farms by the end of the year. The virus subsequently overwintered and has since spread across much of Europe, causing tens of thousands of livestock deaths. In August 2007, BTV-8 reached Great Britain (GB), threatening the large and valuable livestock industry. A voluntary vaccination scheme was launched in GB in May 2008 and, in contrast with elsewhere in Europe, there were no reported cases in GB during 2008.

**Methodology/Principal Findings:**

Here, we use carefully parameterised mathematical models to investigate the spread of BTV in GB and its control by vaccination. In the absence of vaccination, the model predicted severe outbreaks of BTV, particularly for warmer temperatures. Vaccination was predicted to reduce the severity of epidemics, with the greatest reduction achieved for high levels (95%) of vaccine uptake. However, even at this level of uptake the model predicted some spread of BTV. The sensitivity of the predictions to vaccination parameters (time to full protection in cattle, vaccine efficacy), the shape of the transmission kernel and temperature dependence in the transmission of BTV between farms was assessed.

**Conclusions/Significance:**

A combination of lower temperatures and high levels of vaccine uptake (>80%) in the previously-affected areas are likely to be the major contributing factors in the control achieved in England in 2008. However, low levels of vaccination against BTV-8 or the introduction of other serotypes could result in further, potentially severe outbreaks in future.

## Introduction

Amongst the numerous diseases of ruminants, bluetongue (BT) has gained considerable importance in recent years as one of the best examples of the effects of climate change on disease spread [1], [2], [3], [4]. As an arbovirus, bluetongue virus (BTV), the aetiological agent of BT, depends almost entirely on the presence of competent *Culicoides* biting midges to be transmitted to the local host population. Currently, 24 serotypes of BTV have been identified worldwide, with most infected countries confronted with the challenge of dealing with multiple serotypes circulating in their ruminant populations [5]. The global range of BTV has historically been assumed to be restricted by regional differences in vector competence amongst *Culicoides* species as well as by the temperature requirements of the virus for replication. In recent years, however, the emergence and rapid spread of previously unreported serotypes (and strains) has occurred in a number of regions globally, including Europe and North America [5], [4].

Between 1998 and 2006, several BTV serotypes made incursions into Europe, but their distribution was limited to the Mediterranean Basin where the main Afro-Asiatic vector, *Culicoides imicola*, was present [3]. Limited evidence indicated that northern European *Culicoides* species could also potentially transmit the virus [6], but the risk of BT for the northern parts of Europe was considered minimal [7]. Indeed, before 2006, no case of BT had ever been reported above 50°N [1], [2]. It was therefore unexpected, when, in August 2006, the first cases of BTV serotype 8 (BTV-8) were recorded near Maastricht in the Netherlands (over 900km further north than ever before), with subsequent cases reported in Belgium, Germany, France and Luxembourg [8], [9]. By mid-January 2007, approximately 2,000 holdings had been affected. In May 2007, BTV-8 re-emerged and caused major outbreaks across the previously-affected countries, and spread into new areas of northern mainland Europe. The number of BT cases recorded in 2007 reached tens of thousands of affected farms in some countries [9]. The first case of BTV-8 in Great Britain (GB) was reported at Baylham Farm, near Ipswich on 22 September 2007 and, by the end of the year, a total of 67 affected holdings had been identified, although pre-movement testing later increased the number of known affected holdings to 125 by early 2008 [10].

A voluntary vaccination programme was launched in GB in May 2008, making use of an inactivated vaccine against BTV-8 which had only just become available. In contrast with other European countries, GB reported no cases of transmission of BTV-8 within its borders in 2008. It is essential to investigate why this was the case and, in particular, to identify key factors influencing the apparent success of vaccination in order to assess the risk from future reintroductions if, for instance, the level of vaccine uptake declines or if temperatures are warmer during the period of potential BTV transmission. To address these questions we use a stochastic, spatially-explicit model to describe the spread of BTV within and between farms in GB, which incorporates the effects of temperature on infection dynamics and also integrates the currently available data on BTV epidemiology [11].

## Methods

### Modelling Framework

A complete description of the model for the transmission of BTV within and between farms in GB, including parameter estimation, sensitivity analysis and model validation using data on BTV in GB from 2007, is presented elsewhere [11]; here we provide a summary of the approach used.

#### Within-farm model

A stochastic model, which includes two host species (cattle and sheep) and one vector, was used to describe the spread of BTV within a holding. Parameter estimates were obtained from the published literature, using those applicable to the GB situation wherever possible. Explicit temperature dependence was included for the reciprocal of the time interval between blood meals (related to the biting rate), the vector mortality rate and the extrinsic incubation period. Remaining parameters were set for each farm by sampling from appropriate ranges, except for the duration of viraemia in ruminant hosts where point estimates for the (gamma) distribution parameters were used.

#### Between-farm model

A stochastic, spatially explicit farm-level model with a daily time step was developed to describe spread between farms. Transmission between farms was modelled by a generic transmission kernel, which includes both animal movements and vector dispersal (both active and passive). The probability of transmission depends on the distance between farms (i.e. the kernel) and the species composition of the farms. Parameters for the transmission probability were estimated using outbreak data for northern Europe in 2006. Once a farm acquired infection, the within-farm model was simulated based on the number of cattle and sheep kept on the farm and on local temperatures. The impact of movement restrictions on transmission was incorporated by assuming transmission could occur between farms only if both were in a protection zone (PZ), as declared by the Department for Environment, Food and Rural Affairs (Defra).

#### Initial conditions

The model was initialised with a single infected farm (Baylham Farm, near Ipswich) on 4 August 2007. Six additional farms became infected later (two in Cambridgeshire, three in Kent and one in Sussex), with no demonstrable epidemiological link to the main focus in East Anglia. It has been suggested that these additional cases may have been a result of new introduction events. Accordingly, they were included as additional initial foci of infection in the simulations, based on their location and date of reporting.

#### Simulations

Simulations were run until 3 November 2008, the date when Scotland was declared a PZ to allow vaccination. For each scenario the model was run so that 30 outbreaks (defined as any increase in the number of affected holdings beyond those seeded in the simulations; see above) were generated. Replicates were simulated until the required number (30) of outbreaks had been generated (i.e. the number of replicates was not specified in advance, rather it follows a negative binomial distribution). The number of outbreaks was chosen to give robust results without being prohibitively expensive in terms of computation time.

### Data

The location and the number of sheep and cattle on each holding were obtained from June agricultural survey data for 2006. Hourly temperature records for 2006 and 2007 (see Figure S1) were extracted from the BADC/MIDAS database [12] for the 19 meteorological stations used as inputs to the model (see [11]). For each scenario a single year of data were used for the simulations, with a farm using temperature records from its nearest meteorological station.

### Vaccination

Vaccination was assumed to act by reducing the probability of transmission from vector to host (*b*; i.e. it reduces the probability that an animal acquires infection) and from host to vector (β; to reflect lower virus titres in infected, vaccinated animals). The probabilities decreased linearly over time until full protection was reached, so that,
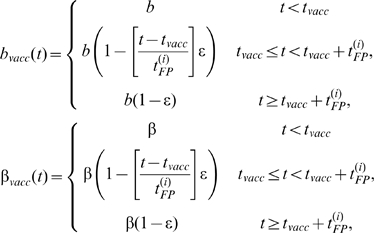
(1)where *b* and β are the baseline probabilities (i.e. in unvaccinated animals), ε is the vaccine efficacy (assumed to be 100%, unless stated otherwise) and *t_vacc_* and 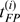 are the time of vaccination and time at which full protection is reached in species *i* (cattle (*C*) or sheep (*S*)), respectively. Data on the time to full protection in sheep and cattle were obtained from information supplied by the vaccine manufacturers: in sheep this is reached at 14 days post vaccination (dpv) (see also [13], [14]); in cattle it is reached at 60 dpv (see also [14]).

Protection zones (PZ), which identify where vaccine can be used, were defined by counties within the vaccination zones declared by Defra. All vaccination scenarios were based on the roll-out plan implemented by Defra [15], [16], using the actual dates of extension of the PZ as published on the Defra website [16] from 30 April 2008 (when the first batch of vaccine was released) up to 1 September 2008 (when the whole of England and Wales was declared to be a PZ). Under this approach farmers in counties not-yet infected were able to vaccinate in a preventive manner.

Only limited data are available on the level of vaccine uptake. In the summer of 2008, farmer surveys were carried out in East Anglia (an area affected by BTV in 2008) by the University of Cambridge, where estimates for vaccine uptake of up to 95% were recorded on sheep farms, although uptake was lower on cattle farms (Webb, Oura, O'Brien, Floyd and JLNW, unpublished). Data on sales of vaccine imply, however, that uptake in other areas was considerably lower. Thus, we simulated vaccination according to the Defra roll-out plan, assuming different levels of uptake (i.e. proportion of farmers vaccinating their stock; Table 1): 95% (the best-case scenario); 80% (the target level of coverage [17]); 50% (a more pessimistic scenario); and a scenario where the level of uptake increased with proximity to the area affected in 2007. In this case, vaccine uptake was 95% in the 2007 PZ, 75% in the 2007 surveillance zone, and 50% elsewhere (the 2007 free area). In addition, the impact of reduced uptake by cattle-only farms was investigated, following preliminary results of surveys conducted in East Anglia in summer 2008, which suggested there was a 10% lower level of uptake in this farm type (Webb, Oura, O'Brien, Floyd and JLNW, unpublished). The different scenarios for vaccine uptake are summarised in Table 1.

**Table 1:** Summary of the scenarios for vaccine uptake.

code	level of uptake (% farms vaccinated)	
	other farms†	cattle-only farms
none	none	none
95	95	95
80	80	80
50	50	50
var	50/75/95‡	50/75/95‡
95LC	95	85
80LC	80	70
50LC	50	40
varLC	50/75/95‡	40/65/85‡

† other farms: mixed cattle and sheep farms and sheep-only farms.

‡ level of uptake in free area/surveillance zone/protection zone, as defined by Defra at the end of 2007.

Under each scenario, a list of farms to be vaccinated within a county was created in the following way. A farm was added to the list with probability given by the specified level of uptake. Once this was done for all farms in the county, the order of vaccination was determined by randomising the list of farms to be vaccinated. The farms on the list were then vaccinated starting from the date when the county became part of a PZ. Because it is farmers themselves who vaccinate their stock, not specialised teams as would be the case for foot-and-mouth disease, vaccination was implemented at a constant number of farms per day in each county, such that all farms were vaccinated within 21 days following the date when that county became part of a PZ. Preliminary results of surveys conducted in East Anglia in summer 2008 by the University of Cambridge indicated that this was an appropriate time-scale to consider (Webb, Oura, O'Brien, Floyd and JLNW, unpublished). If a farm was vaccinated, all animals on that farm were assumed to be vaccinated. The number of doses of vaccine required (one per sheep and two per bovine) was recorded, but was assumed not to constrain the strategy.

### Sensitivity Analysis

Sensitivity analyses were carried out to assess the impact of various model assumptions on the predicted efficacy of a vaccination programme against BTV-8. In particular, we examined the effect of: (i) *Vaccine uptake*: (see above; Table 1); (ii) *Temperature*: The impact of temperature was assessed by using two temperature datasets: 2006 (a warmer year) and 2007 (a cooler year) (see Figure S1); (iii) *Time to full protection in cattle*: The model was simulated assuming full protection in cattle was reached after 30, 45 or 60 dpv; (iv) *Vaccine efficacy*: Values were considered for vaccine efficacy which range from 50% to 100%; (v) *Shape of the transmission kernel*: Four kernels were used to simulate outbreaks: three (Gaussian, exponential and fat-tailed) obtained by fitting models to data on the spread of BTV-8 in northern Europe in 2006 (see [11] for full details), together with that estimated from the 2001 outbreak of foot-and-mouth disease (FMD) in the GB [18], [19]. In addition, the Gaussian kernel (which yielded the best fit to the north European data;[11]) was used with a range of values for its shape parameter; and (vi) *Threshold temperature for between-farm transmission*: Although no evidence for temperature dependence in the transmission of BTV between farms was identified when fitting to the epidemic of BTV-8 in northern European [11], its potential impact was assessed by setting a threshold temperature below which transmission was assumed not occur (none; 5°C; 10°C; and 15°C). A total of 36 scenarios were considered, full details of which are provided in Table S1.

### Statistical Analysis

The results from the simulations were compared to identify factors influencing: (i) the probability that an incursion results in an outbreak of BTV (i.e. there is any increase in the number of affected farms); (ii) the size of the epidemic, expressed as the total number of farms affected during the simulation; and (iii) the probability of extinction (i.e. that an outbreak dies out by the end of the simulation).

The probabilities of an outbreak and of extinction were compared using χ^2^ or Fisher exact tests. Epidemic sizes were compared using generalised linear models (GLM) with negative binomial errors and a log link function. For the combined vaccination and temperature scenarios model construction proceeded by stepwise deletion of non-significant (*P*>0.05) terms starting from an initial model including temperature, vaccine uptake and whether or not uptake was reduced for cattle-only farms as factors, together with pairwise interactions between all factors. For the remaining scenarios, only the factor under consideration (time to full protection, vaccine efficacy, kernel parameter or shape, threshold temperature) was included in the initial model.

## Results

### Probability of an Outbreak

Assuming 2007 temperatures 2.7% (95% confidence interval (CI): 1.9–3.9%) of incursions resulted in outbreaks (cf. [11]). By contrast, a significantly higher proportion of incursions (21.7%; 95% CI: 15.2–29.6%) resulted in outbreaks for the 2006 temperatures (χ^2^ = 95.3, df = 1, *P*<0.001).

### Spatio-Temporal Dynamics of BTV-8 in GB

**Figure 1:**
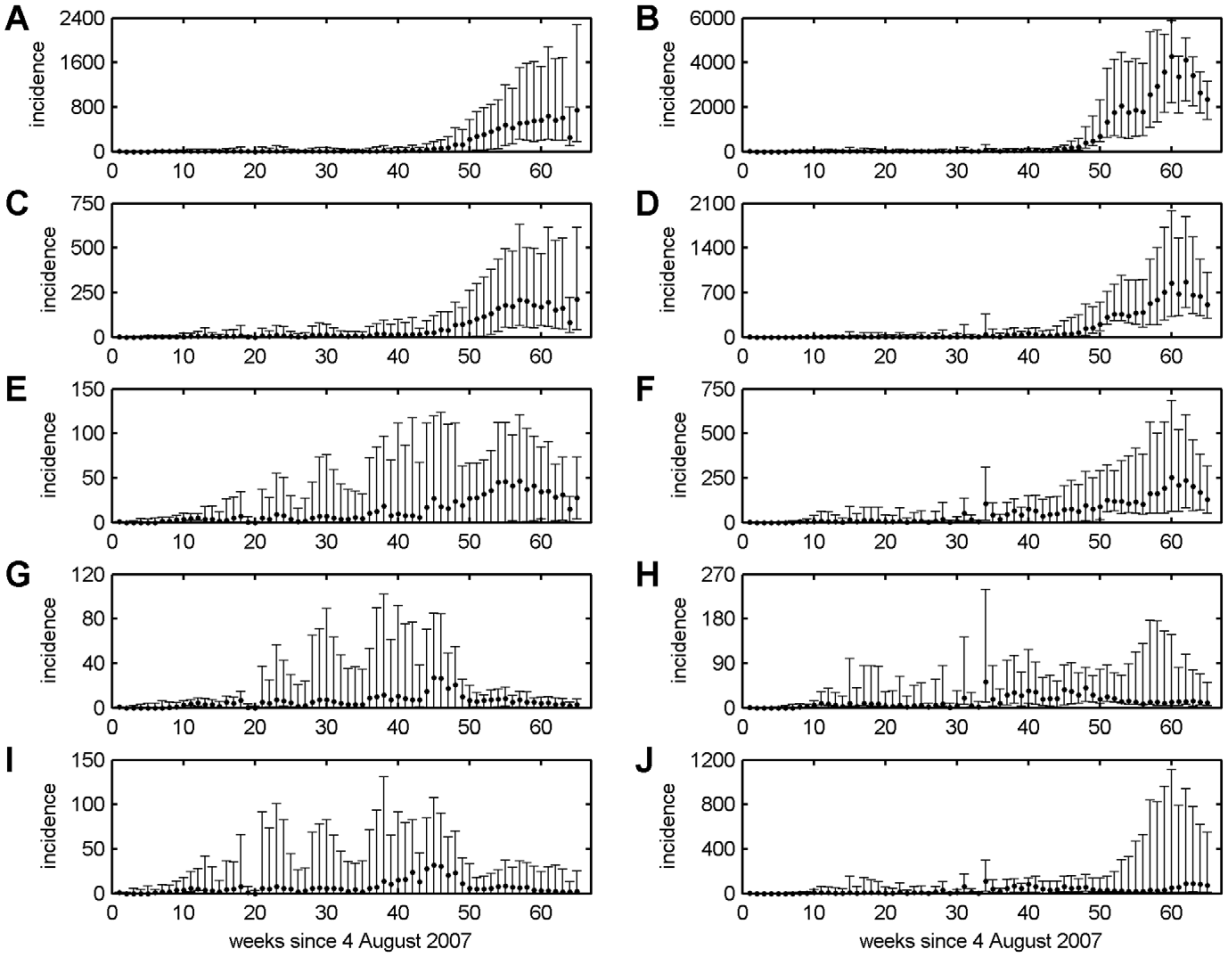
Predicted time-course of BTV-8 epidemics in GB under different vaccination and temperature scenarios. Results presented are for: (A, B) no vaccination; vaccination with (C, D) 50% uptake; (E, F) 80% uptake; (G, H) 95% uptake; and (I, J) variable levels of uptake (95% in the 2007 protection zone, 75% in the 2007 surveillance zone and 50% elsewhere). Simulations are based on (A, C, E, G, I) 2007 or (B, D, F, H, J) 2006 temperatures, assuming the best-fit Gaussian kernel to the north European data, a time to full protection in cattle of 60 dpv and vaccine efficacy of 100%. Each figure shows the median (circles) and 10th and 90th percentiles (error bars) for the incidence of BTV (number of new clinical holdings per week).

**Figure 2:**
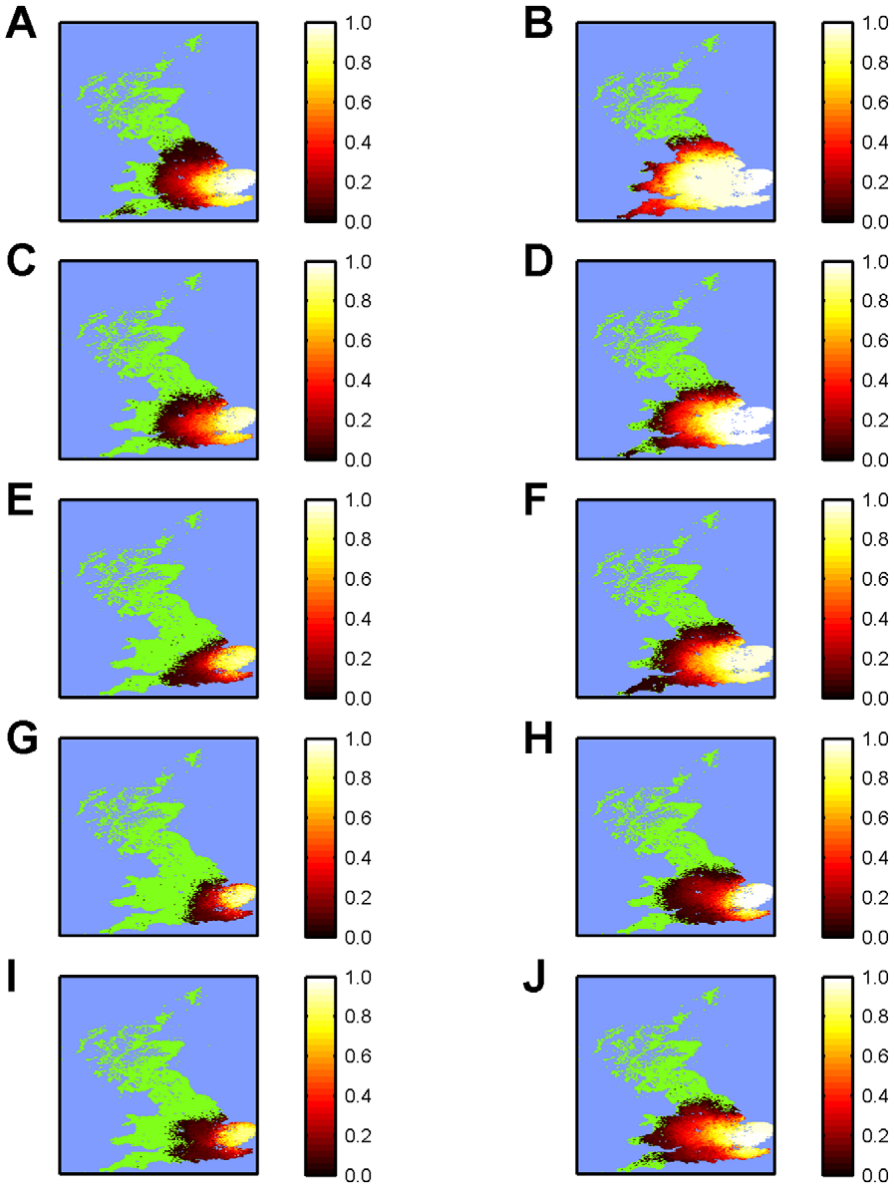
Predicted spatial distribution of BTV-8 in GB under different vaccination and temperature scenarios. The different maps represent the extent of the predicted spatial spread up to the end of 2008 under different scenarios for vaccination and temperature: (A, B) no vaccination; vaccination with (C, D) 50% uptake; (E, F) 80% uptake; (G, H) 95% uptake; and (I, J) variable levels of uptake (95% in the 2007 protection zone, 75% in the 2007 surveillance zone and 50% elsewhere). Simulations are based on (A, C, E, G, I) 2007 or (B, D, F, H, J) 2006 temperatures, assuming the best-fit Gaussian kernel to the north European data, a time to full protection in cattle of 60 dpv and vaccine efficacy of 100%. The maps show the cumulative risk (see colour bars) expressed as the proportion of simulated outbreaks (out of 30 which took off; see Methods) for which at least one farm was affected by BTV within each 5km grid square.

In the absence of vaccination the model predicted severe outbreaks of BTV-8 in GB in terms of both the incidence of clinical disease (Figure 1A,B) and spatial spread (Figure 2A,B), particularly so for simulations using the warmer 2006 temperatures. Vaccination reduced both the incidence and spatial spread, with increasing levels of coverage resulting in greater reductions (Figures 1 and 2). However, even at high levels of uptake (95%) the model predicted some spread of BTV (Figures 1G,H and 2G,H). For the scenarios in which vaccine uptake varied according to distance from the 2007 affected area, the model predictions were similar to those assuming high levels (95%) of uptake everywhere, except for certain replicates where the virus escaped the high uptake area resulting in higher incidence and greater spread (Figures 1I,J and 2I,J; cf. Figures 1G,H and 2G,H). Finally, there was a marked impact of temperature with larger outbreaks (Figure 1) and more extensive spread (Figure 2) for the 2006 compared with the 2007 temperatures.

### Outbreak Size

**Figure 3:**
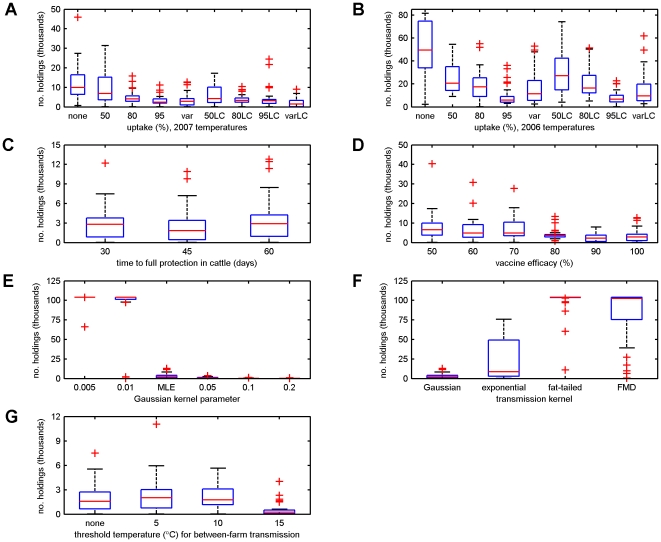
Sensitivity analysis of predicted outbreak size for BTV-8 in GB. Impact of: (A,B) vaccination uptake assuming (A) 2007 or (B) 2006 temperatures (see Table 1 for codes for different uptake levels); (C) time to full protection in cattle; (D) vaccine efficacy; (E) Gaussian kernel parameter; (F) shape of the transmission kernel; and (G) a threshold temperature below which no transmission between farms was assumed to occur. The default scenario assumed variable vaccine uptake, 2007 temperatures, a time to full protection in cattle of 60 dpv, vaccine efficacy of 100% and the best-fit Gaussian kernel to the north European data (i.e. no temperature dependence). The boxplots show the median (red line) and the 25th and 75th percentile (blue box); the whiskers indicate 1.5 times the interquartile range; and the red crosses show outlying values for the cumulative number of affected holdings at 3 November 2008.

Comparing the results of different scenarios for vaccine uptake and temperature (Figure 3A,B) indicated that both vaccine uptake and temperature had a significant (*P*<0.001) impact on outbreak size, but that the effect of uptake depended on the temperature data-set (i.e. there was a significant (*P* = 0.006) interaction between uptake and temperature). However, lower uptake by cattle-only farms did not significantly affect outbreak size (*P* = 0.25).

For those simulations using the 2007 temperature data-set the scenarios could be divided simply into high (≥80%) or low (≤50%) levels of uptake, with smaller outbreaks occurring for the high compared with low levels of uptake (Figure 3A). In contrast, there were differences in outbreak size between all five levels of vaccine uptake considered (Table 1) for simulations based on 2006 temperatures (Figure 3B).

### Probability of Extinction

In only 2.4% (95% CI: 1.3–4.1%) of outbreaks was BTV predicted to die out by the end of the simulations (3 November 2008). There was a difference between the probability of extinction based on the 2006 temperatures (0.4%; 95% CI: 0.01–2.0%) and that based on the 2007 temperatures (4.4%; 95% CI: 2.3–7.6%), which was not significant at the conventional 5% level but was significant at the 10% level (Fisher exact test, *P* = 0.097). However, there were no significant differences in the probability of extinction between levels of vaccine uptake for those simulations using the same temperature data-set (Fisher exact test, *P*>0.5).

### Sensitivity Analysis

The sensitivity of the predicted outbreak size was assessed for various assumptions underlying vaccination (time to full protection in cattle, vaccine efficacy), the transmission kernel (kernel parameter, kernel shape) and temperature dependence in the transmission of BTV between farms (Figure 3C–G). Changing the time to full protection in cattle from 60 days (default) to 30 or 45 days did not significantly (*P* = 0.5) affect the outbreak size (Figure 3C). Outbreak size declined as vaccine efficacy increased (*b* = −0.020, 95% CI: (−0.029, −0.011); *P*<0.001). Moreover, there was evidence for a threshold effect of vaccine efficacy, with outbreaks significantly smaller for high (≥80%) compared with low (≤70%) levels of efficacy (*b* = −0.72, 95% CI: (−1.01,−0.43); *P*<0.001) (Figure 3D). Outbreaks were predicted to become significantly (*P*<0.001) smaller as the Gaussian kernel parameter increased, with a marked change in outbreak size occurring as the parameter changed from 0.01 to 0.034 (Figure 3E). In addition, the shape of the kernel significantly (*P*<0.001) affected the predicted outbreak size, with the smallest outbreaks for the Gaussian kernel, followed by the exponential kernel with the largest outbreaks for the fat-tailed or FMD kernels (Figure 3F). Finally, if a threshold temperature was set below which no transmission occurred between farms, outbreaks were significantly (*P*<0.001) smaller for a threshold of 15°C, but there were no significant differences when there was no threshold or when it was set at 5°C or 10°C (Figure 3G).

## Discussion

This paper presents an investigation of the key factors influencing the success of a vaccination programme against BTV and, in particular, the effects of the level of vaccine uptake by farmers and temperature. To address these issues we used mathematical models developed to describe the spread of BTV within and between farms in GB, which had been validated using data on the spread of BTV-8 in GB in 2007 [11].

Qualitatively, the model predicts a similar pattern of outbreaks (Figure 1) to that observed in northern Europe in 2006 and 2007 (i.e. in the absence of vaccination), with small numbers of affected holdings in the first year, followed by large numbers in the second year [9]. Although a quantitative comparison between countries in the number of outbreaks is problematic because of differences in size and farming practices, outbreaks involving many thousands of BTV-8 affected holdings were reported in 2007 for Belgium, Germany, France and the Netherlands [9]. This was also the case for the simulations for GB using the 2007 temperature data (Figure 3A), though outbreak sizes were much larger for simulations using the 2006 temperatures (Figure 3B).

Vaccination was predicted to reduce incidence (Figure 1), extent of spread (Figure 2) and outbreak size (Figure 3A,B), with a greater reduction achieved for higher levels of coverage, in particular, in previously affected areas. However, even at high levels of uptake, there was still some spread, though at much lower levels compared with no vaccination (Figure 1), which is, in part, a consequence of transmission occurring in the model before vaccination started (on 30 April 2008, i.e. 39 weeks after 4 August 2007). This observation suggests more consideration needs to be given to the seasonal dynamics of the vector [11] and, more generally, to the overwintering of BTV, something which is discussed in more detail below.

A high level (>80%) of vaccine uptake in the previously-affected area was most important for controlling the spread of BTV (Figures 1 and 2). If, however, uptake was lower in areas outside those previously affected, there was a risk of escape of BTV into areas with lower coverage and, hence, greater potential for spread (Figure 2I,J; cf. Figure 2G,H). This observation highlights the need for a buffer zone beyond the previously affected area with high levels of vaccine uptake to contain the spread of BTV. The size of the buffer will depend critically on the transmission kernel [11], with a larger buffer required for kernels with fatter tails (i.e. higher probabilities of transmission at long distances).

Our analyses suggest that the absence of reported cases of BTV-8 in GB during 2008 and the apparent success of control can be attributed to the high levels of vaccine coverage achieved in previously affected areas, coupled with cooler temperatures in 2008. Although there were no reported cases in GB, the virus was still circulating in northern mainland Europe in 2008 and 2009 and, consequently, there remains a risk of reintroduction. Furthermore, other serotypes currently threaten GB, including BTV-1, which reached the northern French coast in late 2008 with further cases in 2009. In addition, BTV-6 was reported to be circulating in the Netherlands and Germany in 2008 [20], and evidence of BTV-11 infection (but not circulation) was found in cattle in Belgium [21].

Although an inactivated vaccine against BTV-1 is available, it cannot currently be used in a preventive manner in GB without the prior creation of a so-called “blue zone” for that serotype. In addition, no vaccine has yet been developed against BTV-6 or BTV-11. The results of the simulations indicate that a re-incursion by BTV-8 or an incursion by BTV-1 (or any other serotype) could be controlled provided high levels of vaccine coverage are achieved (Figures 1 & 2). As re-vaccination with the new inactivated vaccines is recommended annually, it is essential that the level of vaccination against BTV-8 does not decline over the coming year and that sufficient stocks of vaccine against BTV-1 are available and can be deployed rapidly should an incursion of this serotype occur. By contrast, with no inactivated vaccine available, an incursion of BTV-6 could potentially be very serious, especially if temperatures are warmer (Figures 1 and 2), though this assumes that the transmission dynamics for different serotypes would be similar.

The model predictions for the impact of vaccination were not greatly affected by assumptions made about the time to full protection in cattle (Figure 3C). This is largely a consequence of the way in which vaccine was deployed in areas well in advance of BTV arriving. If, however, the vaccine were being used reactively in response to a new incursion, this parameter could influence the success of vaccination. As would be expected, a decrease in vaccine efficacy results in an increase in outbreak size (Figure 3D). Although a linear relationship between the logarithm of the outbreak size and the efficacy adequately captured the model predictions, there was evidence for a threshold effect, with a stepped change in outbreak size at efficacies between 70–80% (Figure 3D). This is a consequence of herd immunity [22], such that a herd is protected (i.e. the basic reproduction number (*R*_0_) is reduced below one) provided that a large enough proportion of animals in the herd is protected. Estimates for *R*_0_ for BTV [23] suggest that this should be achieved with a vaccine efficacy of at least 75%, which is likely to be the case, at least for sheep [13].

The shape of the transmission kernel had a major impact on the model predictions (Figure 3E,F). Increasing the probability of longer-range spread, whether by decreasing the shape parameter or by using a kernel with fat tails (i.e. fat-tailed or FMD), dramatically increases the size of the outbreak, even with vaccination. This reflects the impact of movement restrictions (which were assumed to prevent transmission outside the PZ, so that long range transmission is principally a result of animal movements) on predicted spread using different kernels [11]. For the Gaussian kernel, movement restrictions had only a small effect on spread, whereas for the exponential and, especially, the fat-tailed and FMD kernels, these restrictions greatly reduced spread. For these kernels, the roll-out of the protection zone to allow vaccination (see Vaccination in Methods section) has the effect of markedly increasing the potential for transmission and, hence, for spread into areas with low vaccine uptake.

In our earlier analyses of outbreak data for BTV-8 in northern Europe during 2006, we did not identify a significant impact of temperature on transmission [11]. By contrast, the sensitivity analyses in the present study indicated that temperature dependence in the spread of BTV between farms (in this case via a threshold temperature below which transmission was assumed not to occur) could impact on model predictions for the spread and control of BTV. In particular, if no transmission was assumed to occur below 15°C, outbreaks were significantly smaller than if there was no threshold or the threshold was ≤10°C (Figure 3G). A threshold temperature is a simple, but crude and potentially inaccurate method of including temperature dependence. For example, *Culicoides* species are known to be active at temperatures below 15°C [24], while animal movements are unlikely to be influenced by ambient temperatures. Accordingly, a more detailed description of transmission between farms is needed which disentangles animal from vector movements and which accounts for seasonal variation in vector activity.

Under all the scenarios considered in this paper, BTV-8 was predicted to have persisted in a majority of replicates. The long-term dynamics of BTV-8 are of considerable importance, both in terms of determining how long vaccination needs to be carried out (annual booster vaccination is required to maintain protection) and when freedom from virus circulation can begin to be assessed. However, investigating the long-term dynamics of BTV requires a greater understanding of the ability of the virus to overwinter [25] and of the biology of those *Culicoides* species implicated as vectors in northern Europe [7].

Recent evidence of vertical [26], [27], [28], [29] and horizontal [28] transmission in the vertebrate host have re-launched the debate about alternative transmission mechanisms being involved in the spread of BTV. These mechanisms could provide routes by which the virus can overwinter in temperate climates [25]; the recent epidemic in northern Europe having confirmed that BTV-8 is able to overwinter successfully and with apparent high probability. The outbreak in northern Europe has also demonstrated the potential for Palaearctic species of *Culicoides* to transmit BTV efficiently. However, these species are not yet fully characterised in terms of their life history parameters or their responses to the prevailing environmental conditions; vector competence, host preference, biting habits and abundance of the different vector species are also largely unknown. While potential differences between vector species could have a major impact on the risk of BTV to British livestock, the fact that BTV-1 has spread in northern Europe underlines the capacity of the northern species of *Culicoides* to transmit other serotypes and strains of the virus.

This study represents a first step in analysing the spread and control of BTV at a national level, but shows that the model provides a suitable framework for investigating the consequences of BTV incursions to GB and assessing the relative efficacy of different options for control. Future work should focus on incorporating in the model more realistic descriptions of the seasonal vector dynamics and of the overwintering mechanisms for BTV.

## Supporting Information

Table S1 Full list of scenarios considered for the spread and control of bluetongue virus (BTV) serotype 8 in Great Britain (GB).
(0.08 MB PDF)

Figure S1 Hourly temperature records for 2006 and 2007 for 19 meteorological stations used as inputs to the model for the transmission of bluetongue virus within and between farms. Data for 2006 (blue) and 2007 (red) are plotted for each meteorological station shown in order from the southernmost to the northern most station.
(1.29 MB TIF)
