# Supplementary material for: The Spread of Bluetongue Virus Serotype 8 in Great Britain and Its Control by Vaccination
Source: PLoS One. 2010 Feb 22;5(2):e9353. doi: 10.1371/journal.pone.0009353 (PMC2825270; doi:10.1371/journal.pone.0009353)
Supplement: Table S1 — Full list of scenarios considered for the spread and control of bluetongue virus (BTV) serotype 8 in Great Britain (GB). (0.08 MB PDF) [file pone.0009353.s001.pdf]

Table S1. Full list of scenarios considered for the spread and control of bluetongue virus (BTV) serotype 8 in Great Britain (GB).

scenario	vaccine uptake (%)	reduced uptake in cattle?	temperature data-set	time to full protection in cattle (dpv)	vaccine efficacy (%)	kernel shape	kernel parameter(s)[‡]
1	none	no	2007	60	100	Gaussian	MLE
2	95	no	2007	60	100	Gaussian	MLE
3	80	no	2007	60	100	Gaussian	MLE
4	50	no	2007	60	100	Gaussian	MLE
5	variable[†]	no	2007	60	100	Gaussian	MLE
6	95	yes[*]	2007	60	100	Gaussian	MLE
7	80	yes[*]	2007	60	100	Gaussian	MLE
8	50	yes[*]	2007	60	100	Gaussian	MLE
9	variable[†]	yes[*]	2007	60	100	Gaussian	MLE
10	none	no	2006	60	100	Gaussian	MLE
11	95	no	2006	60	100	Gaussian	MLE
12	80	no	2006	60	100	Gaussian	MLE
13	50	no	2006	60	100	Gaussian	MLE
14	variable[†]	no	2006	60	100	Gaussian	MLE
15	95	yes[*]	2006	60	100	Gaussian	MLE
16	80	yes[*]	2006	60	100	Gaussian	MLE
17	50	yes[*]	2006	60	100	Gaussian	MLE
18	variable[†]	yes[*]	2006	60	100	Gaussian	MLE
19	variable[†]	no	2007	30	100	Gaussian	MLE
20	variable[†]	no	2007	45	100	Gaussian	MLE
21	variable[†]	no	2007	60	50	Gaussian	MLE
22	variable[†]	no	2007	60	60	Gaussian	MLE
23	variable[†]	no	2007	60	70	Gaussian	MLE
24	variable[†]	no	2007	60	80	Gaussian	MLE
25	variable[†]	no	2007	60	90	Gaussian	MLE
26	variable[†]	no	2007	60	100	Gaussian	0.005
27	variable[†]	no	2007	60	100	Gaussian	0.01

scenario	vaccine uptake (%)	reduced uptake in cattle?	temperature data-set	time to full protection in cattle (dpv)	vaccine efficacy (%)	kernel shape	kernel parameter(s)[‡]
28	variable[†]	no	2007	60	100	Gaussian	0.05
29	variable[†]	no	2007	60	100	Gaussian	0.1
30	variable[†]	no	2007	60	100	Gaussian	0.2
31	variable[†]	no	2007	60	100	exponential	MLE
32	variable[†]	no	2007	60	100	fat-tailed	MLE
33	variable[†]	no	2007	60	100	FMD	MLE

[†] variable uptake: 50% in free area; 75% in surveillance zone; and 95% in protection zone, as defined by Defra at the end of 2007 (see table 1 in the main paper)

[*] uptake by cattle-only farms is 10% lower than by other farm types (i.e. sheep-only or mixed cattle and sheep farms; see table 1 in the main paper)

[‡] MLE: kernel parameters are the maximum likelihood estimates based on fitting to data on the north European outbreak of BTV in 2006 (Gaussian, exponential, fat-tailed; see Szmaragd et al. 2009 *PLoS ONE* **4**, e7741) or to the 2001 epidemic of foot-and-mouth disease (FMD) in GB (see Chis Ster & Ferguson 2007 *PLoS ONE* **2**, e502; Chis Ster et al. 2009 *Epidemics* **1**, 21-34).
