# Supplementary figures and images for: The Spread of Bluetongue Virus Serotype 8 in Great Britain and Its Control by Vaccination
Source: PLoS One. 2010 Feb 22;5(2):e9353. doi: 10.1371/journal.pone.0009353 (PMC2825270; doi:10.1371/journal.pone.0009353)

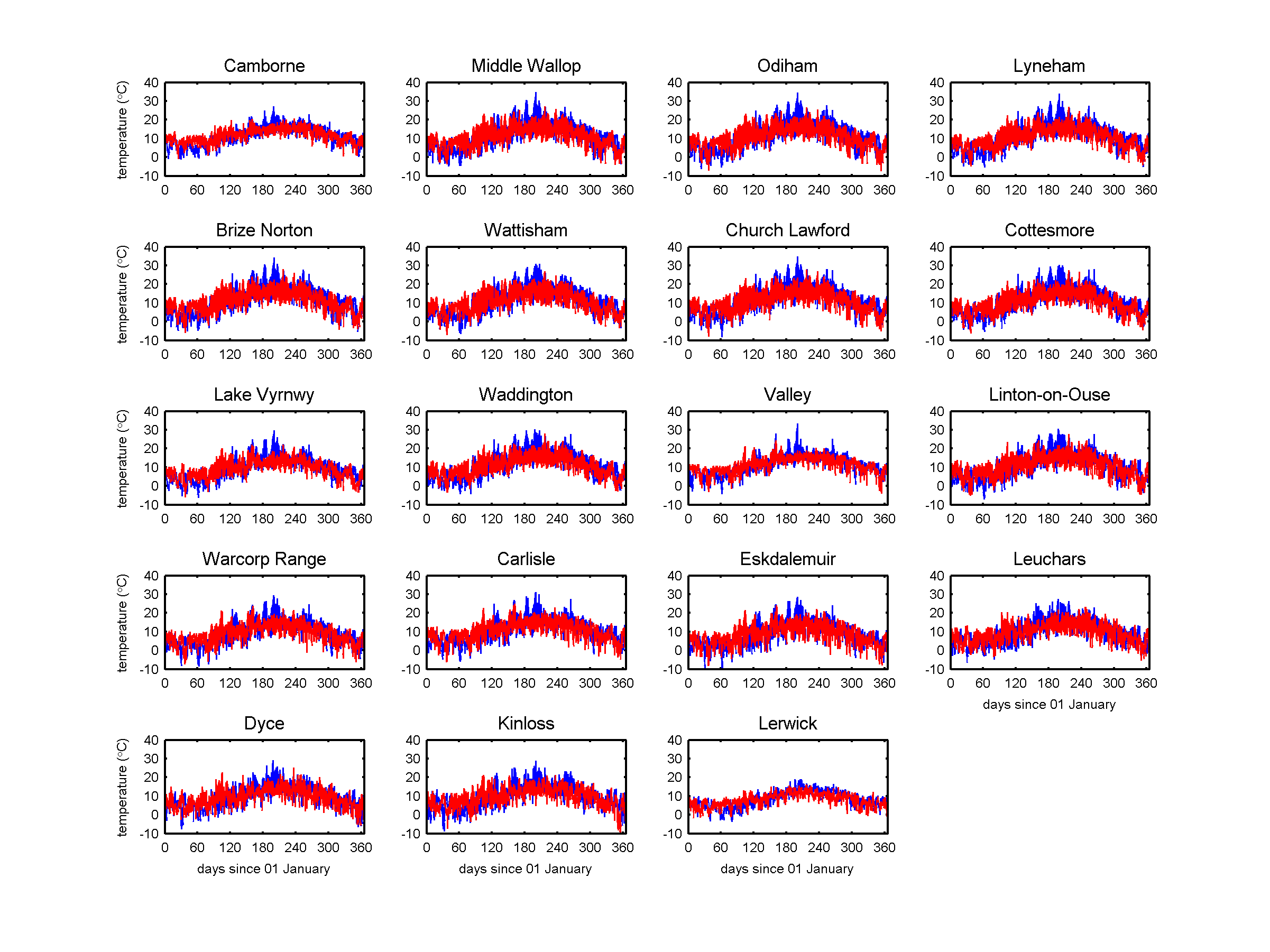

Supplement: Figure S1 — Hourly temperature records for 2006 and 2007 for 19 meteorological stations used as inputs to the model for the transmission of bluetongue virus within and between farms. Data for 2006 (blue) and 2007 (red) are plotted for each meteorological station shown in order from the southernmost to the northern most station. (1.29 MB TIF) [file pone.0009353.s002.tif]
